# MiR-205-5p Functions as a Tumor Suppressor in Gastric Cancer Cells through Downregulating FAM84B

**DOI:** 10.1155/2022/8267891

**Published:** 2022-05-27

**Authors:** Xi Chen, Lei Zhang, JingBo Geng, Zhong Chen, XiaoPeng Cui

**Affiliations:** ^1^Department of Gastrointestinal Surgery, Affiliated Hospital of Nantong University, Nantong, Jiangsu, China; ^2^Department of General Surgery, Affiliated Hospital of Nantong University, Nantong, Jiangsu, China

## Abstract

MicroRNAs (miRNAs) participate in the formation of multiple diseases, including gastric cancer (GC), through modulating specific targets. Here, we explored the functions and regulatory mechanisms of miR-205-5p in GC. MiR-205-5p levels were detected in GC cells through qRT-PCR. Besides, the role of miR-205-5p in cell proliferation, cell apoptosis, cell cycle, cell invasion, and metastasis was assessed through CCK-8 assay, colony formation, flow cytometry, scratch assay, transwell, and western blot. Moreover, the Starbase website was used to predict the target gene of miR-205-5p, further verified by a dual-luciferase reporter assay. Furthermore, the functional effects of the family with sequence similarity 84 member B (FAM84B) on GC mediated by miR-205-5p upregulation were further investigated. MiR-205-5p expression was decreased in GC cells. Upregulation of miR-205-5p inhibited cell proliferation and metastasis and induced apoptosis and cycle arrest of GC cells. Moreover, FAM84B was predicted and confirmed as a target of miR-205-5p and negatively related to miR-205-5p. Mechanically, FAM84B overexpression partially rescued the functional effects of miR-205-5p upregulation on GC cell progression. This study suggests the potential of miR-205-5p/FAM84B as novel targets for the treatment of GC.

## 1. Introduction

Gastric cancer (GC) is one of the most dangerous cancers worldwide with high morbidity and mortality [1]. Because of the great progress in the diagnosis and treatment, the long-term survival of GC patients has been remarkably prolonged in the past few decades. However, many patients have developed into advanced stages at the time of diagnosis due to lack of early symptoms. For GC patients with advanced stages of metastatic tumors, the therapeutic effects were disappointing with less than a 30% 5-year survival rate [2]. Therefore, identifying effective early diagnostic biomarkers and treatment targets is the main task for GC control and prevention.

MicroRNAs (miRNAs) are nucleotides with a length of about 18–25 bp and do not encode proteins. MicroRNAs are closely involved in the regulation of posttranscriptional gene expression. Numerous evidence report that the expression profile of miRNAs was remarkably abnormal in tumors and played protumor or antitumor effects according to the function of their target genes. Recently, it was suggested that miRNAs were involved in the regulation of tumor cells' malignant phenotype, such as proliferation, apoptosis, differentiation, migration, and invasion. Several miRNAs have been implicated in GC progression, including miR-21-5p, miR-214, miR-143, miRNA-17-3p, and miRNA-17-5p [3–6]. For example, miR-21-5p promotes GC cells proliferation through modulating SMAD7 [3]. MiR-182 levels were reduced in GC patients. MiR-182 overexpression inhibited GC progression via targeting KLF4 [7]. Moreover, miR-205-5p was expressed at a low level in certain cancers, including renal carcinoma [8], prostatic carcinoma [9], breast cancer [10], colorectal cancer [11], and ovarian cancer [12]. MiR-205-5p inhibits angiogenesis in GC by attenuating the expression of VEGFA and FGF1 [13]. But the precise impacts of miR-205-5p on influencing GC progression and the related mechanisms remained largely unknown.

This work focused on elucidating the biological functions of miR-205-5p on GC progression by examining its expression in GC cells and identifying its target.

## 2. Materials and Methods

### 2.1. Cell Culture and Transfection

Human gastric epithelial cell line GES-1 and GC cells (AGS, SGC-7901, SNU-1, and MGC-803) were provided by the American Type Culture Collection (ATCC, USA) and cultured within DMEM (Thermo Fisher Scientific, USA) containing 10% FBS (Thermo Fisher Scientific, USA) at 37°C and 5% CO_2_.

MiR-205-5p mimic, Lv-FAM84B, and negative controls were obtained from Ambion (Austin, USA) and transfected into GC cells (5 × 10^5^) maintained within the 6-well plates using Lipofectamine 3000 (Invitrogen, USA). Transfection efficiency was examined by qRT-PCR after 48 h.

### 2.2. qRT-PCR

After extracting total RNA from cells, cDNA was prepared with the total RNA by the RNeasy plus micro kit through reverse transcription in line with specific instructions, as the starting material of qRT-PCR carried out using step one system (Life Technologies Corp). Sequences of all primers were designed by a Primer Premier software 4.0 (Premier, Canada) and as Table 1. U6 or GAPDH were normalized by the 2^−ΔΔCT^ approach [14].

### 2.3. CCK-8 Assay

Transfected GC cells (5 × 10^3^/well) were inoculated into 96-well plates. After incubation for 24, 48, and 72 h, the CCK-8 kit (Sigma, USA) was utilized. A microplate reader (Tecan Infinite M200, Switzerland) was used to detect the absorbance (OD) value of 490 nm.

### 2.4. Colony Formation Assay

Transfected GC cells (1 × 10^3^/well) were cultured in 6-well plates, and the medium was replaced every 2–3 days for a total of two weeks. Later, GC cells were stained using 1% crystal violet for 30 min and imaged (Nikon, Japan).

### 2.5. Flow Cytometry

To analyze cell apoptosis, the culture medium of GC cells from different groups was collected. Then Annexin V-FITC and PI were added for 10 min. Flow cytometry was conducted for measuring apoptotic cells. For the cell cycle, GC cells from different groups were harvested, followed by overnight fixation with 70% EtOH and 30 min of PI staining. Finally, flow cytometry was adopted for detecting cell cycle distribution.

### 2.6. Western Blot Analysis

Protein was isolated from GC cells and measured through the BCA kit (Beyotime Biotechnology, China). Protein was separated by 10% SDS-PAGE, and it was extracted and then shifted into PVDF membranes (Millipore, USA). Next, membranes were incubated using 5% skimmed milk, followed by overnight incubation with primary antibodies including anti-cyclin D1 (1 : 2, 000, 26939-1-AP, Proteintech, China), anti-CDK2 (1 : 2, 000, 10122-1-AP, Proteintech, China), anti-P21 (1 : 2, 000, 10355-1-AP, Proteintech, China), anti-MMP2 (1 : 2, 000, 10373-2-AP, Proteintech, China), anti-MMP9 (1 : 2, 000, 10375-2-AP, Proteintech, China), anti-COX-2 (1 : 2, 000, 27308-1-AP, Proteintech, China), and anti-*β*-actin (1 : 5, 000, 66009-1-Ig, Proteintech, China), with *β*-actin being the endogenous control under 4°C. Furthermore, they were incubated for 1 h using HRP-labeled secondary antibody (1 : 4, 000, SA00004-10, Proteintech, China) under ambient temperature. Finally, the enhanced chemiluminescence kit (ECL, Millipore, Bedford, USA) was utilized to observe protein blots, whereas an ImageJ software (NIH, version 4.3) was adopted for quantification.

### 2.7. Scratch Assay

Transfected cells (5 × 10^5^/well) were plated into 6-well plates. After cells reached 80% confluence, a wound was scratched. Cell images at 0 and 48 h were captured with a light microscope (Nikon, Japan) (200×) [15].

### 2.8. Transwell Assay

For invasion assays, Matrigel was dissolved with serum-free DMEM, and uniformly covered in the Transwell chamber for 1 h at 37°C. For migration and invasion assays, GC cells from different groups were seeded into upper chambers at the appropriate density. Medium containing 10% FBS was added into lower chambers. After 48-h incubation at a room temperature of 37^o^C, migrated and invaded cells were stained with crystal violet (0.1%) and captured (Nikon, Japan) (200×).

### 2.9. Dual Luciferase Reporter Assay

PmirGLO dual-luciferase vector (150 ng) (Promega, USA) was subcloned with FAM84B WT/MUT for generating pmirGLO-FAM84B WT/MUT. Then, miR-205-5p mimic and pmirGLO-FAM84B WT/MUT or NC mimic were cotransfected for 48 h, and the dual-luciferase reporter system (Promega, USA) was utilized.

### 2.10. Statistical Analysis

Data were analyzed using a GraphPad Prism 5.0 and presented as mean ± SD. Differences between the groups were compared by ANOVA as well as Tukey's poc host analysis. *p* < 0.05 was considered statistically significant.

## 3. Results

### 3.1. MiR-205-5p Overexpression Suppressed GC Cells Proliferation and Metastasis

**Figure 1:**
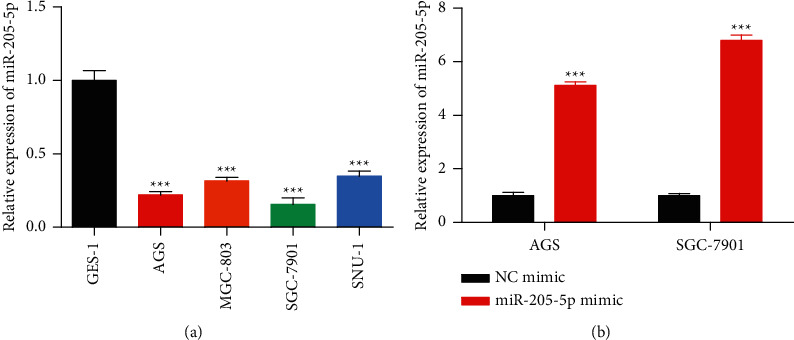
MiR-205-5p was downregulated in gastric cancer cells. (a) qRT-PCR on the expression of miR-205-5p in AGS, MGC-803, SGC-7901, and SNU-1 cells and normal gastric epithelial cells-1 (GES-1). (b) NC mimic and miR-205-5p mimic were transfected into GC cells by using Lipofectamine 2000 and transfection efficiencies were detected by qRT-PCR. ^*∗∗∗*^*p* <0.001 *vs*. GES-1 or NC mimic. All experiments were performed in triplicate and repeated at least three times.

QRT-PCR was used to examine the miR-205-5p expression in GC cells and it was found that miR-205-5p was expressed at a low level in GC cells, especially in AGS and SGC-7901 cells (Figure 1(a)). The miR-205-5p mimic was transfected into GC cells, and transfection efficiency is shown in Figure 1(b).

**Figure 2:**
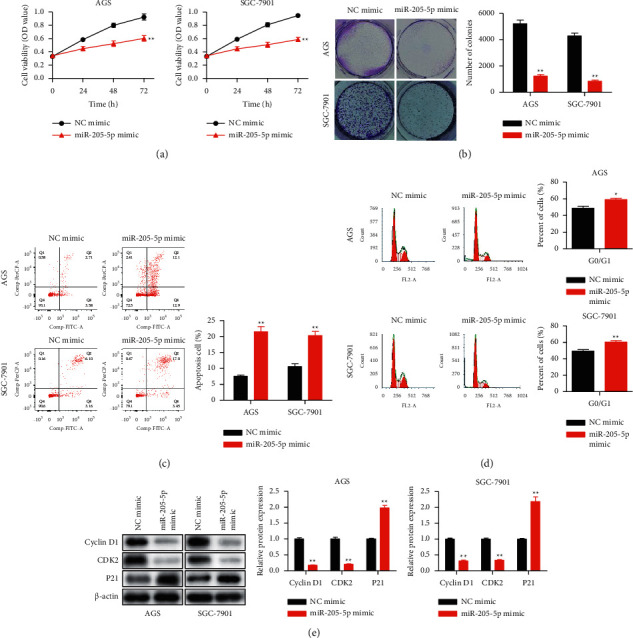
Overexpression of miR-205-5p inhibited cell proliferation and induced cell apoptosis and cell cycle arrest in GC. NC mimic and miR-205-5p mimic were transfected into AGS and SGC-7901 cells by using Lipofectamine 2000. (a) Cell viability of transfected AGS and SGC-7901 cells detected by using the CCK-8 assay. (b) Colony formation on transfected AGS and SGC-7901 cells. (c) Cell apoptosis and (d) cell cycle of transfected AGS and SGC-7901 cells detected by flow cytometry. (e) The protein expression of cyclin D1, CDK2, and P21 detected in transfected AGS and SGC-7901 cells by western blot. ^*∗*^*p* <0.05, ^*∗∗*^*p* <0.01 and ^*∗∗∗*^*p* <0.001 *vs*. NC mimic. All experiments were performed in triplicate and repeated at least three times.

Then CCK-8 and colony formation were conducted for assessing the effect of miR-205-5p mimic on GC cells viability and proliferation. As shown in Figures 2(a) and 2(b), miR-205-5p upregulation decreased the viability and colony-forming ability of GC cells. In addition, flow cytometry was conducted for measuring cell apoptosis and cell cycle. Data in Figures 2(c) and 2(d) indicated that miR-205-5p overexpression promoted apoptosis and induced cell cycle arrest at the G0/G1 phase of GC cells. Furthermore, a western blot assay was employed for determining the effect of miR-205-5p mimic on cell cycle-related protein levels. Based on Figure 2(e), miR-205-5p overexpression decreased the protein expressions of cyclin D1 and CDK2 but increased that of P21.

**Figure 3:**
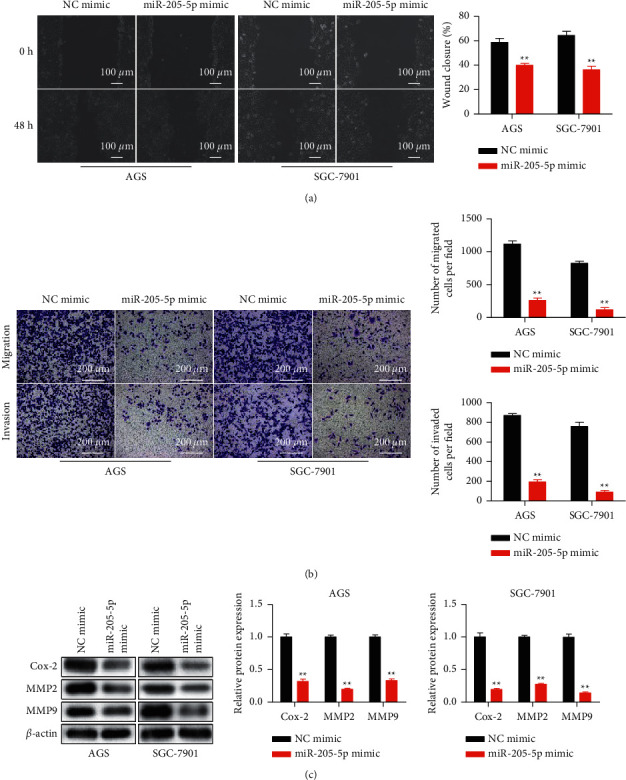
Overexpression of miR-205-5p inhibited cell migration and invasion in GC. NC mimic and miR-205-5p mimic were transfected into AGS and SGC-7901 cells by using Lipofectamine 2000. (a) Cell migration of transfected cells detected by a scratch assay. (b) Transwell assay on the migration and invasion activities of transfected cells. (c) The protein expression of COX-2, MMP2, and MMP9 detected in transfected AGS and SGC-7901 cells by western blot. ^*∗∗*^*p* <0.01 *vs*. NC mimic. All experiments were performed in triplicate and repeated at least three times.

Moreover, scratch and transwell analyses were carried out for assessing miR-205-5p overexpression on metastasis in GC cells. Figures 3(a) and 3(b) showed that miR-205-5p high-expression decreased the abilities of GC cell metastasis. Furthermore, the expression of metastasis-related proteins was also detected. As shown in Figure 3(c), miR-205-5p mimics repressed the expression of MMP2, MMP9, and COX-2. These data indicated that miR-205-5p overexpression repressed GC cells proliferation and metastasis.

### 3.2. MiR-205-5p Targeted FAM84B in GC

**Figure 4:**
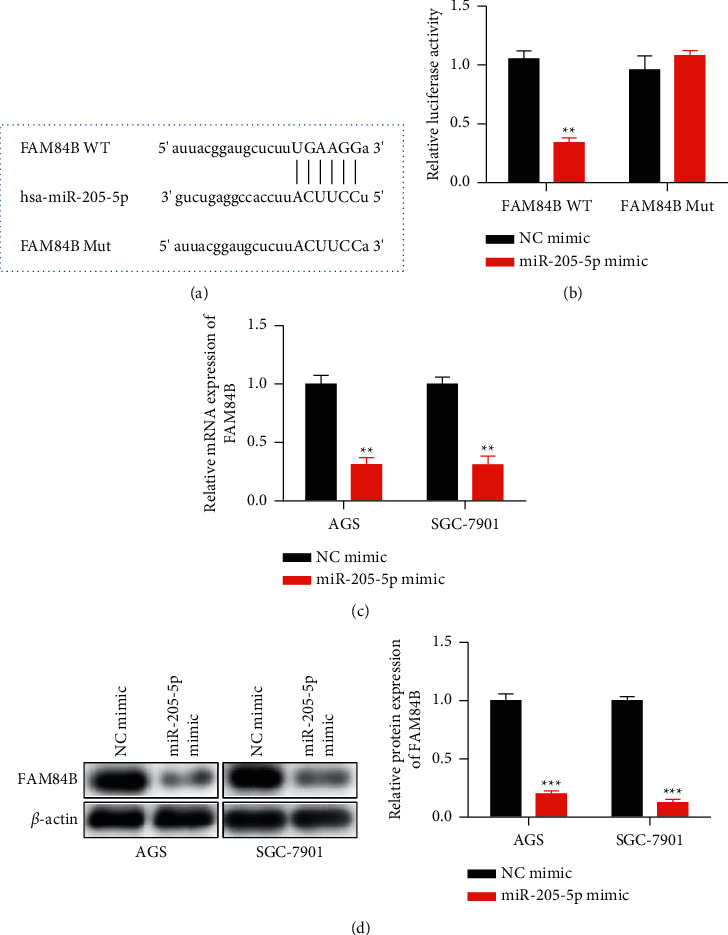
MiR-205-5p bound to FAM84B and inhibited its expression. (a) The binding sites between miR-205-5p and FAM84B 3′ untranslated region (3′ UTR) were predicted on the Starbase website (http://starbase.sysu.edu.cn/index.php). (b) A dual-luciferase reporter assay was performed to confirm the direct interaction between miR-205-5p and FAM84B 3′ UTR. (c) NC mimic and miR-205-5p mimic were transfected into AGS and SGC-7901 cells by using Lipofectamine 2000. The expression of FAM84B mRNA was detected by qRT-PCR. (d) NC mimic and miR-205-5p mimic were transfected into AGS and SGC-7901 cells by using Lipofectamine 2000. The expression of the FAM84B protein was detected by western blot. ^*∗∗*^*p* <0.01 and ^*∗∗∗*^*p* <0.001 *vs*. NC mimic. All experiments were performed in triplicate and repeated at least three times.

MiRNAs regulated gene expression through binding to the 3′ UTR of target genes and inducing degradation of their mRNAs [16]. Bioinformatics tool (Starbase) was used to search for the possible miR-205-5p targets and FAM84B was found to be the most related gene. The dual-luciferase reporter analysis verified the target relationship (Figures 4(a) and 4(b)). Moreover, FAM84B levels in GC cells transfected with miR-205-5p mimic were detected through qRT-PCR and western blot. Data in Figures 4(c) and 4(d) showed that miR-205-5p overexpression caused FAM84B downregulation in GC cells. These data indicated that FAM84B was the target of miR-205-5p in GC.

### 3.3. FAM84B Overexpression Reversed the Antitumor Effect of MiR-205-5p Upregulation in GC Cells

**Figure 5:**
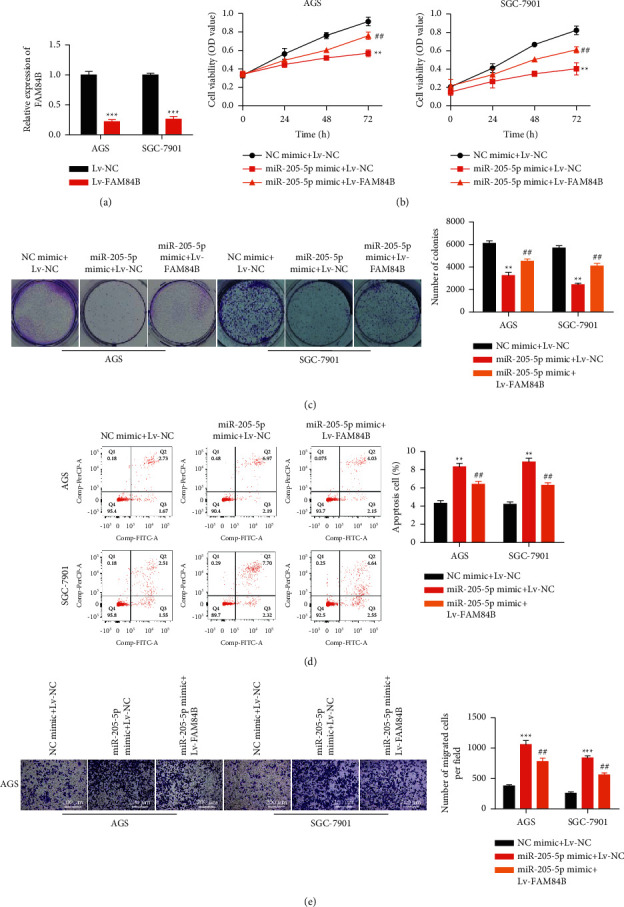
FAM84B overexpression reversed antitumor effects of miR-205-5p in GC. The experiments were performed in three groups: (1) NC mimic + Lv-NC; (2) miR-205-5p mimic + Lv-NC; and (3) miR-205-5p mimic + Lv-FAM84B. (a) The recombinant lentivirus vectors that contained FAM84B full sequence or NC sequence were transfected into AGS and SGC-7901 cells, and FAM84B expression was detected by qRT-PCR. ^*∗∗∗*^*p* <0.001 *vs*. Lv-NC (b) Cell viability of transfected AGS and SGC-7901 cells was detected by using the CCK-8 assay. (c) Colony formation of transfected AGS and SGC-7901 cells was detected. (d) Cell apoptosis of transfected AGS and SGC-7901 cells was detected by flow cytometry. (e) Transwell assay investigated the migration and invasion activities of transfected cells. ^*∗∗*^*p* <0.01 and ^*∗∗∗*^*p* <0.001 *vs*. NC mimic; ^*∗∗*^*p* <0.01 *vs*. MiR-205-5p mimic + Lv-NC. All experiments were performed in triplicate and repeated at least three times.

For illustrating the role of miR-205-5p in regulating GC progression via targeting FAM84B, miR-205-5p mimic and/or Lv-FAM84B were transfected into GC cells (Figure 5(a)). According to CCK-8 and colony formation assays, FAM84B upregulation partially restored the inhibitory effect of miR-205-5p mimic on GC cell proliferation (Figures 5(b) and 5(c)). In addition, FAM84B overexpression partially rescued the promoting influences of miR-205-5p mimic on GC cell apoptosis (Figure 5(d)). Moreover, based on Figure 5(e), Lv-FAM84B partially restored the suppressive effects of miR-205-5p mimic on GC cell metastasis. These data indicated that FAM84B upregulation partially restored the impacts of miR-205-5p mimic on the biological functions of GC cells.

## 4. Discussion

Our data elucidated a novel molecular mechanism by which miR-205-5p acted as a tumor suppressor in GC cells through targeting FAM84B and inhibiting its expression. Evidences have revealed that miRNAs exert essential roles on GC tumorigenesis, development, and chemoresistance, and were associated with patients' characteristics and outcomes. For example, miRNA-194 was associated with good prognosis in GC patients and induced GC cell growth inhibition and cell cycle arrest [17]. Moreover, miR-3664-5p suppressed the proliferation and metastasis of GC by negatively modulating MTDH through attenuating NF-*κ*B signaling pathway [18]. In addition, miRNA-192 and miR-215 enhanced the growth and migration of GC cells through targeting APC [19]. Therefore. miRNAs were involved in multiple malignant phenotypes of GC through regulating differential gene expression. Here, we identified that miR-205-5p expression was decreased in GC cells. Particularly, miR-205-5p is expressed at low level and function as a tumor suppressor in multiple cancer types. Guo et al. found miR-205-5p was downregulated in gallbladder cancer (GBC). MiR-205-5p upregulation repressed drug resistance, proliferation, and promoted apoptosis of GBC stem cells [20]. In a recent study found that up-regulation of miRNA-205-5p exerted an antitumor effect accomplished by decreasing VEGFA and inactivating PI3K/Akt/mTOR in renal carcinoma cells [8]. However, in lung cancer, miRNA-205-5p was upregulated in tumor tissues and promoted cell proliferation and survival through modulating erbB3 [21]. The differential function of miRNA-205-5p might be due to the differences in the molecular microenvironment in cancer types. Since the targets of miRNAs varied in different cell types and disease contexts [22, 23], it is necessary to identify the function of miRNA-205-5p on other cancer types.

MiRNA-205-5p upregulation suppressed the proliferation, metastasis, induced cell cycle arrest, and apoptosis of GC cells accompanied by decreasing cyclin D1, CDK2, and increasing P21. Cyclin D1, acts as a regulatory subunit of cyclin-dependent kinase (CDK4 or CDK6), which is necessary for G1 to S progression in the cell cycle [17]. Cyclin D1 is upregulated or mutated in various tumor types, which alters cell proliferation [24]. CDK2 is also a cell cycle checkpoint protein, which controls G1 to S phase transition and forms complex with cyclin A or E [25, 26]. P21 functions as a cyclin-dependent kinase inhibitor, which binds to CDK2 or CDK4 [27, 28]. It is widely known that P21 was the important target protein of tumor suppressor p53, through which this protein mediated p53-dependent cell cycle arrest [29, 30]. In summary, cyclin/CDK were the important downstream signal proteins in the antitumor function of miRNA-205-5p. Tumor metastasis, driven by cancer cell invasion and migration, was one of the most important causes for GC-related deaths [2]. Here, we demonstrated that miRNA-205-5p overexpression led to significant inhibition of GC cell metastasis by downregulating COX-2, MMP2, and MMP9 levels. COX-2, as a member of COX family, is closely related to tumorigenesis and invasiveness [31]. Matrix metalloproteinases (MMPs) are positive regulators of intestinal tumorigenesis [32].

FAM84B, known for its potential connections with Myc and Ras, is located at the chromosomal locus 8q24.21 [33]. The amplification of the 8q24.21 region had been found in various cancer types, including GC [34], ovarian cancer [35], prostate cancer [36], and others. Currently, the oncogenic function of Myc has been widely demonstrated, while few studies report similar results for FAM84B. Recently, the elevation of FAM84B expression has been reported in esophageal squamous cell carcinoma [37] and prostate cancer [38]. It is worth noting that FAM84B promoted tumor growth and metastasis in prostate cancer via activation of the AKT signal pathway [39]. However, whether FAM84B plays a role in GC progression remains unclear. We found that FAM84B was the target of miR-205-5p, and its overexpression could reverse the antitumor effect of miR-205-5p upregulation.

Taken together, our study showed that miR-205-5p was decreased, and FAM84B increased in GC cells. Upregulation of miR-205-5p inhibited GC cell proliferation and metastasis, and promoted GC cell apoptosis and cell cycle arrest by regulating FAM84B. Our results suggested that miR-205-5p could be a potential target for the GC treatment.

## Figures and Tables

**Table 1:** The primer sequences for qRT-PCR.

Gene name	Forward	Reverse
MiR-205-5p	5′-TCCTTCATTCCACCGGAGTCTG-3	5′-GCGAGCACAGAATTAATACGAC-3′
FAM84B	5′-GACCCACCTAAGTTACAAGGAAG-3′	5′-GTAGAACACGGAGCATTCCAC-3′
U6	5′-CTCGCTTCGGCAGCACA-3′	5′-AACGCTTCACGAATTTGCGT-3′
GAPDH	5′-CCTCGTCTCATAGACAAGATGGT-3′	5′-GGGTAGAGTCATACTGGA ACATG-3′

## Data Availability

All data generated or analyzed during this study are included in this published article.
